# Haemodynamics of hyperthyroidism: increased cardiac work and findings related to vasodilatation

**DOI:** 10.1530/ETJ-24-0090

**Published:** 2024-10-24

**Authors:** Nelli Suonsyrjä, Saara Metso, Eeva Moilanen, Jukka Mustonen, Pia Jaatinen, Ilkka Pörsti

**Affiliations:** 1 Faculty of Medicine and Health Technology, Tampere University, Tampere, Finland; 2 Department of Internal Medicine, Tampere University Hospital, Tampere, Finland; 3 Immunopharmacology Research Group, Tampere University and Tampere University Hospital, Tampere, Finland

**Keywords:** haemodynamics, hyperthyroidism, impedance cardiography, pulse wave analysis

## Abstract

**Objective:**

Hyperthyroidism increases cardiovascular morbidity and mortality, but the underlying mechanisms are not fully understood. In this study, we compared non-invasive haemodynamics between 20 hyperthyroid patients and 60 euthyroid subjects.

**Methods:**

The measurements were performed median 6 days after the initiation of antithyroid medication when the patients were still hyperthyroid. Three controls matched for age, sex, body mass index, and smoking status were selected for each patient. Recordings were performed during rest and passive head-up tilt using whole-body impedance cardiography, radial pulse wave analysis, and finger blood pressure measurements.

**Results:**

Systolic and diastolic blood pressures in the aorta and radial artery were similar in hyperthyroid and euthyroid subjects, while finger blood pressure was 16/12 mm Hg lower in hyperthyroidism (*P* < 0.001). Pulse wave velocity and aortic pulse pressure were similar, but radial pulse pressure was ~5 mm Hg higher in hyperthyroidism (*P* = 0.040) due to augmented amplification (*P* = 0.045). Systemic vascular resistance was reduced (−18%), whereas heart rate (+19 beats/min), cardiac index (+28%), and left cardiac work (+31%) were increased in hyperthyroidism (*P* < 0.001). Subendocardial viability ratio, reflecting the balance between coronary perfusion and pressure load, was reduced by 19% in hyperthyroidism (*P* < 0.001). Compared with euthyroid subjects, hyperthyroid patients presented with reductions in systolic and diastolic finger blood pressures (*P* < 0.001), and a higher increase in heart rate (*P* = 0.014) during upright posture.

**Conclusions:**

Hyperthyroid patients exhibited hyperdynamic circulation, reduced vascular resistance, reduced peripheral but not central blood pressure, and higher pulse pressure amplification. Furthermore, the left cardiac workload was increased in parallel with unfavourable changes in coronary perfusion conditions.

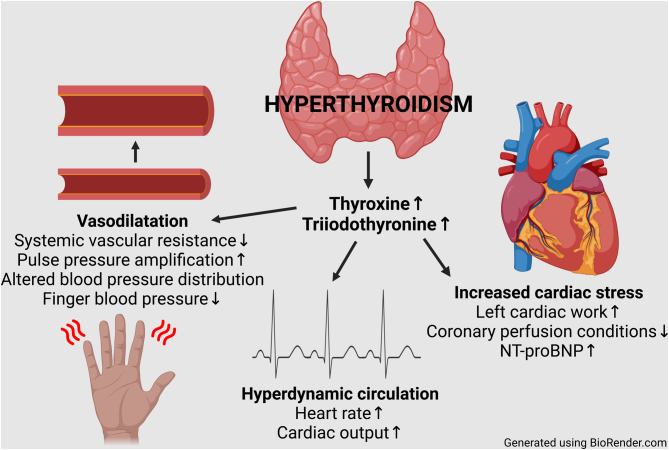

## Introduction

Thyroid hormones have significant influences on the regulation of the cardiovascular system, and cardiovascular manifestations are characteristic of thyroid hormone excess (1). Hyperthyroidism is associated with an increased risk of cardiovascular morbidity and mortality, which may remain elevated for several years after the restoration of normal thyroid status (2, 3, 4, 5). Whether hyperthyroidism causes irreversible cardiovascular damage remains unknown, and the mechanisms underlying the cardiovascular risks have not been fully clarified.

During hyperthyroidism, the cardiovascular system responds to the increased metabolic rate and heat production, leading to hyperdynamic circulation (1, 5, 6). Thyroid hormones, specifically triiodothyronine (T3), regulate cardiac myocytes and vascular smooth muscle cells through genomic and non-genomic actions. These actions decrease systemic vascular resistance and enhance the inotropy, chronotropy, and diastolic function of the heart (1, 5, 6). Activation of the renin-angiotensin-aldosterone system, increased blood volume, and enhanced erythropoietin secretion have been reported in hyperthyroid subjects (1, 5, 6). These factors, along with increased myocardial contractility, likely contribute to the high cardiac output observed in hyperthyroidism (1, 5, 6).

Physical challenge may reveal changes in cardiovascular function not evident at rest (7, 8). Exercise intolerance is common in hyperthyroidism, but comprehensive information about upright haemodynamics during hyperthyroidism is lacking (1). The transition from a supine to an upright posture induces several haemodynamic changes, and the physical challenge it presents can be regarded as a stress test for cardiovascular regulation (7, 9). Comprehensive recording of haemodynamic variables and evaluation of the cardiovascular response to an upright position have not been studied in hyperthyroid subjects. Only a few studies have assessed central haemodynamics and the interrelationship between various haemodynamic variables in hyperthyroidism, yielding conflicting results (10, 11, 12, 13). In this cross-sectional study, we examined cardiovascular function during the supine position and passive head-up tilt to achieve a comprehensive view of the haemodynamic changes associated with hyperthyroidism and the potential cardiovascular alterations contributing to elevated cardiovascular morbidity in hyperthyroid individuals.

## Methods

### Study subjects

Subjects referred due to a newly diagnosed hyperthyroidism to the endocrinology outpatient clinic of Tampere University Hospital during the years 2016–2019 were recruited to an ongoing DYNAMIC study examining haemodynamics using non-invasive methods (14, 15, 16). The hyperthyroid patients were invited to participate in the study. Hyperthyroidism was defined as a low thyroid-stimulating hormone (TSH) level combined with elevated free thyroxine (fT4) and/or free triiodothyronine (fT3) concentrations (17). According to the regional clinical practice, antithyroid drug therapy (ATD) was initiated in primary health care at the time of diagnosis. The haemodynamic measurements were performed with a minimum delay before a significant response to the ATD therapy was achieved (see below). If a beta blocker was prescribed for hyperthyroid symptoms, it was paused 3 days prior to the measurements to avoid masking of the haemodynamic alterations of hyperthyroidism (18).

For each hyperthyroid participant, three euthyroid controls matched for age, sex, BMI, and smoking status were chosen from participants of our prospective follow-up study (DYNAMIC) on non-invasive haemodynamics. The control subjects were recruited by announcements in local newspapers and from the clients of Varala Sports Institute, the staff of Tampere University Hospital and Tampere University, and patients treated at Tampere University Hospital and nearby occupational health care units (14, 15, 16).

All participants were interviewed and examined by a physician and information on lifestyle, medical and family history history were gathered. Weekly alcohol consumption was evaluated as the number of drinks (12 g absolute alcohol) consumed, and physical activity as the number of ≥30 min exercise periods. The exclusion criteria were acute illness other than hyperthyroidism, inability to pause beta-blocker treatment, heart rhythm other than sinus rhythm, coronary artery disease, heart failure, active kidney or liver disease, a psychiatric condition beyond mild depression or anxiety, or a history of alcohol or substance misuse.

All participants signed an informed consent form. The study adhered to the Declaration of Helsinki, and the study protocol was approved by the Ethics Committee of the Pirkanmaa Hospital District (R06086M) and registered in 2006 in Clinicaltrialsregister.eu (Eudra-CT 2006-002065-39) and in 2012 in ClinicalTrials.gov (NCT01742702).

### Laboratory analyses

Blood and urine samples were taken after about 12 h of fasting. An electro-chemiluminescence immunoassay (Elecsys Cobas e immunoassay analyser, ECLIA; Roche Diagnostics) was used to determine the plasma TSH, fT4, fT3, and thyroid peroxidase antibody (TPOAb). Thyrotropin receptor antibodies (TRAbs) were analysed by a fluoroenzymeimmunoassay (EliA anti-TSH-R method, Phadia AB). The reference ranges were as follows: TSH 0.27–4.2 mU/L, fT4 11.0–22.0 pmol/L, fT3 3.1–6.8 pmol/L. Two reference ranges for TRAb were applied (<1 IU/L or <2.9 IU/L) depending on the time of the measurement. The normal range for TPOAb was less than 34 kU/L. Plasma N-terminal pro-B-type natriuretic peptide (NT-proBNP) and N-terminal pro-atrial natriuretic peptide (NT-proANP) were analysed by enzyme-linked immunosorbent assays (NT-proBNP ELISA, Abcam; NT-ProANP DuoSet ELISA, R&D Systems Europe Ltd, Abingdon, UK). Other laboratory analyses were performed as previously described (19).

### Haemodynamic measurements

The haemodynamic recording was performed by a trained research nurse using tonometric pulse wave analysis and whole-body impedance cardiography as previously described (14, 15, 20). Smoking, caffeine-containing products, and heavy meals were to be avoided for ≥4 h, and alcohol and strenuous exercise for ≥24 h prior to the recording. Impedance cardiography electrodes were placed on the body surface, a tonometric sensor on the left radial artery pulsation, an oscillometric brachial cuff on the right upper arm, and a photoplethysmographic sensor on the 3rd left finger. The left arm was set in support to 90 degrees of abduction, positioning the wrist sensor at the heart level both supine and upright. Haemodynamics were recorded for 5 min supine and for 5 min during passive head-up tilt to 60 degrees (14, 15). The repeatability and reproducibility of the protocol have been reported (14, 15). Routine 12-lead electrocardiograms were recorded, and the Cornell voltage-duration product was calculated.

#### Pulse wave analysis

A tonometric sensor (Colin BP-508T, Colin Medical Instruments Corp., San Antonio, TX, USA) captured the pulse waveform and blood pressure (BP) from the left radial artery. The device was calibrated approximately every 2.5 min by contralateral brachial BP measurements (14, 16). Commerial software (SphygmoCor PWMx, AtCor Medical, West Ryde, NSW, Australia) generated the aortic pulse waveform from the radial signal and calculated aortic BP, aortic pulse pressure (PP), augmentation index (AIx, augmented pressure/pulse pressure × 100), AIx adjusted to heart rate 75/min (AIx@75), aortic reflection time, and Buckberg subendocardial viability ratio (SEVR) (21). BP was also recorded from the left 3rd finger (Finometer^®^, Finapres Medical Systems, Enschede, the Netherlands).

#### Whole-body impedance cardiography

A CircMon^®^ device (JR Medical Ltd., Tallinn, Estonia) registered heart rate (HR) and body electrical impedance during cardiac cycles to evaluate stroke volume, cardiac output, pulse wave velocity (PVW), and extracellular water (ECW) volume (22, 23, 24). Systemic vascular resistance was determined from cardiac output and radial BP (25). Stroke volume, cardiac output, and systemic vascular resistance were indexed to body surface area (DuBois equation) and presented as stroke index (SI), cardiac index (CI), and systemic vascular resistance index (SVRI). The left cardiac work index (LCWI) was calculated by the formula 0.0143 × (mean aortic pressure – assumed normal pulmonary artery occlusion pressure) × cardiac index (18). PWV was adjusted for mean aortic pressure according to the recommendations (26, 27). The reliability of CircMon^®^–derived stroke volume and cardiac output measurements have been demonstrated by comparing the results with echocardiography (15), thermodilution, and the direct oxygen Fick method (22). PWV measured by CircMon^®^ correlates well with tonometric and ultrasound methods (24, 25).

### Statistics

The mean values of the haemodynamic variables from each minute were calculated and used in statistics. The results in the figures are presented as means and standard errors of the mean. Continuous variables presented in Table 1 were compared with the unpaired t-test for normally distributed variables and with the Mann–Whitney *U* test for non-normally distributed variables and presented as mean and standard deviation (s.d.) or median and (25th–75th percentile). The *χ*2 test was used to compare categorical variables. The generalized estimated equations (GEE) were used to analyse differences in the haemodynamic variables between hyperthyroid and control subjects and to evaluate the effect of hyperthyroidism, posture, and their interaction on the variables. In hyperthyroid subjects, the correlations between fT4, fT3, and haemodynamic variables were evaluated using the Spearman correlation coefficient (r_S_). IBM SPSS Statistics Version 28 (IBM) was used, and *P* value < 0.05 was considered statistically significant.

**Table 1:** Clinical characteristics of the study subjects. Results shown as mean (standard deviation) or median [25th–75th percentile] according to the distribution of the variable.

	Control	*n*	Hyperthyroidism	*n*	*P*
Age, years	45.9 (35.7–53.3)	60	48.8 (34.6–53.5)	20	0.859
Sex, male (%)	9 (15%)	60	3 (15%)	20	1.000
Weight (kg)	68.0 (61.5–78.5)	60	69.5 (55.5–81.0)	20	0.802
Height (cm)	168.2 (7.1)	60	166.3 (7.9)	20	0.325
BMI (kg/m^2^)	24.2 (22.0–27.0)	60	25.7 (20.9–29.9)	20	0.575
Smoking					0.227
Never	53.3%	32	40%	8	
Present	13.3%	8	30%	6	
Previous	33.3%	20	30%	6	
Alcohol (drinks/week)	2.0 (0–4)	56	1 (0-1.5)	20	0.091
Office SBP, seated (mmHg)*	127 (116–151)	59	131 (115–140)	20	0.648
Office DBP, seated (mmHg)*	84 (12)	59	82 (9)	20	0.543
Office heart rate (bpm)*	67 (8.8)	56	86 (12.6)	20	<0.001
Laboratory SBP, supine (mmHg)^†^	126 (112–140)	60	129 (110–137)	20	0.755
Laboratory DBP, supine (mmHg)^†^	78 (11)	60	69 (8)	20	0.003
Cornell voltage duration product (ms*mm)	1715 (1306–2045)	60	1590 (1382–1779)	20	0.371
Extracellular water volume (L)	12.0 (1.4)	59	11.5 (2.0)	20	0.237

*measured by physician; ^†^measured by research nurse.

BMI, body mass index; BP, blood pressure; DBP, diastolic blood pressure; SBP, systolic blood pressure.

## Results

### Study population

Twenty hyperthyroid and 60 controls were included. The median duration of ATD therapy preceding the hemodynamic recordings was 6 days (1–11 days). The median age of the hyperthyroid subjects was 48.8 years (range: 26–69 years) and most of them (85%, *n* = 17) were female (Table 1). Ninety percent (*n* = 18) of the patients had positive TRAbs and 55% (*n* = 11) had positive TPOAbs. During haemodynamic recordings, the TSH level was ≤ 0.01 mU/L in most of the hyperthyroid subjects (90%, *n* = 18) and median free T4 and free T3 values were 25.0 mU/L (20.8–38.8) and 10.2 mU/L (7.3–13.2), respectively (Table 2).

**Table 2:** Thyroid function tests and antibodies in the hyperthyroid patients. Results shown as median (25th -75th percentile) or as *n* (%)

	Hyperthyroidism	*n*
TSH, mU/L	0.01 (0.01–0.01)	20
Free T4 at diagnosis, pmol/L	37.4 (29.5–62.5)	20
Free T4*, pmol/L	25.0 (20.8–38.8)	20
Free T3*, pmol/L	10.2 (7.3–13.2)	20
TRAb positive (%)	18 (90)	20
TRAb (IU/L)	6.3 (4.7–12.5)	18
TPOAb positive (%)	11 (55)	20
TPOAb (kU/L)	235 (127–554)	11

T3, triiodothyronine; T4, thyroxine; TPOAb, thyroid peroxidase antibody; TRAb, thyrotropin receptor antibody; TSH, thyroid-stimulating hormone.

*during measurements

There were no differences in age, sex distribution, BMI, smoking status, or alcohol use between the hyperthyroid and the control subjects (Table 1). In auscultatory measurements, seated office BPs did not differ between the groups, but the heart rate was higher in hyperthyroidism. In auscultatory supine measurements, systolic BPs did not differ, whilst diastolic BP was lower among hyperthyroid subjects. Cornell voltage duration product and extracellular water volume were similar in the groups (Table 1).

Blood haemoglobin, and plasma concentrations of creatinine, parathyroid hormone, total cholesterol, HDL cholesterol, and creatinine+cystatin C-based estimated GFR were lower, while plasma sodium, cystatin C, calcium, triglycerides, and glucose concentrations, and HOMA-IR were higher among hyperthyroid subjects than in controls (Table 3). No differences were observed in blood leukocyte count, and in plasma C-reactive protein, potassium, LDL cholesterol or insulin concentrations, or in the 24-h excretion of sodium or potassium. Plasma NT-ProANP was similar in both groups, but NT-ProBNP was higher in hyperthyroid patients than in controls (Table 3).

**Table 3:** Laboratory data of the study subjects. Results shown as mean (standard deviation) or median (25th -75th percentile) according to the distribution of the variable.

	Control	*n*	Hyperthyroidism	*n*	*P*
Haemoglobin (g/L)	139 (11.3)	60	133 (12.1)	20	0.039
Leukocyte count (1*10^9^/L)	5.8 (4.8–6.6)	60	5.5 (4.8–7.3)	20	0.718
C-reactive protein (mg/L)	0.6 (0.5–1.6)	60	1.2 (0.5–4.5)	20	0.156
Potassium (mmol/L)	3.7 (3.6–3.7)	60	3.8 (3.6–3.9)	20	0.064
24-h urine potassium excretion (mmol)	76 (29)	59	67 (25)	17	0.265
Sodium (mmol/L)	140 (2.3)	60	142 (1.9)	20	0.002
24-h urine sodium excretion (mmol)	140 (94–176)	57	124 (92–156)	17	0.644
Creatinine (μmol/L)	68 (61–74)	60	47 (44–54)	20	<0.001
Cystatin C (mg/L)	0.74 (0.66–0.88)	60	1.12 (0.97–1.34)	20	<0.001
Estimated GFR (ml/min/1.73m^2^)*	102 (95–112)	60	90 (83–99)	20	<0.001
Calcium (mmol/L)	2.26 (0.09)	60	2.41 (0.09)	20	<0.001
Parathyroid hormone (pmol/L)	4.5 (1.4)	60	2.3 (0.8)	20	<0.001
Total cholesterol (mmol/L)	4.9 (4.2–5.4)	60	3.6 (3.3–4.4)	20	<0.001
Triglycerides (mmol/L)	0.93 (0.61–1.30)	60	1.23 (0.99–1.64)	20	0.023
HDL cholesterol (mmol/L)	1.80 (0.48)	60	1.38 (0.32)	20	0.001
LDL cholesterol (mmol/L)	2.6 (2.0–3.1)	60	2.1 (1.9–2.7)	20	0.119
Glucose (mmol/L)	5.2 (4.9–5.6)	60	6.0 (5.3–6.30)	20	0.001
Insulin (mU/L)	6.2 (4.2–11.9)	60	8.6 (5.6–14.7)	20	0.096
HOMA-IR (units)	1.40 (0.98–2.80)	60	2.34 (1.36–4.00)	20	0.031
NT-ProANP (ng/mL)	3.46 (1.96–5.06)	60	3.20 (2.35–5.02)	20	0.872
NT-ProBNP (pg/mL)	54.9 (42.3–95.3)	56	107.4 (47.1–405.7)	19	0.015

*GFR, glomerular filtration rate based on combined cystatin C and creatinine CKD-EPI formula (48)

HOMA-IR, homeostatic model assessment of insulin resistance; NT-ProANP, N-terminal pro-atrial natriuretic peptide; NT-ProBNP, N-terminal pro B-type natriuretic peptide.

The use of medications (Supplementary Table 1, see section on supplementary materials given at the end of this article) or the prevalence of hypertension, diabetes, or dyslipidaemia did not differ between hyperthyroid and control subjects (data not shown). The hyperthyroid subjects had the following medical conditions: primary hypertension (*n* = 3), type 2 diabetes (two), asthma (two), coeliac disease (two), migraine (two), hypercholesterolaemia (two), inflammatory skin disease (one), and glomerulonephritis in total remission (one); the control subjects had primary hypertension (*n* = 13), hyperlipidaemia (ten), asthma (two), hypothyroidism (four), type 2 diabetes (one), operatively treated aortic coarctation (one), prostate hyperplasia (one), migraine (four), and previously treated breast cancer (one).

### Haemodynamic measurements

**Figure 1:**
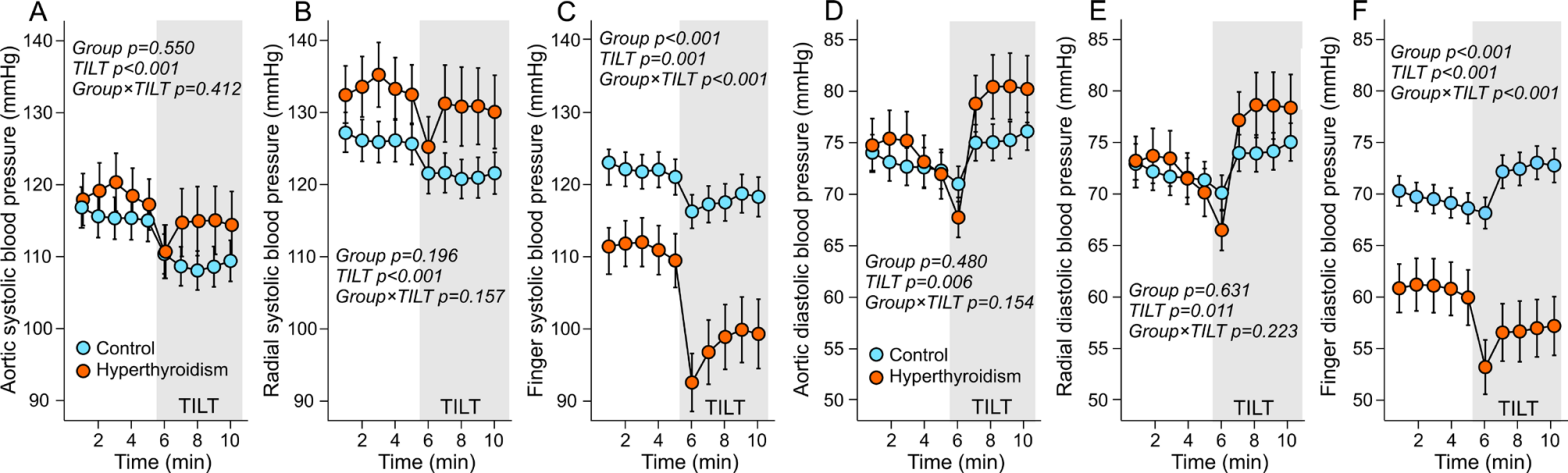
Systolic and diastolic blood pressure in the aorta (A and D), radial artery (B and E), and third finger (C and F) during supine position and passive head-up tilt in 20 patients with hyperthyroidism and in 60 euthyroid controls. Statistics by generalized estimating equations.

Aortic and radial artery systolic and diastolic BP were similar in hyperthyroid and control subjects, and in response to head-up tilt systolic BP decreased and diastolic BP increased in both groups (Fig. 1A, B, D and E). In the finger measurements, however, both systolic and diastolic BPs were lower in hyperthyroid patients (Fig. 1C and F). Furthermore, significant interactions with posture were found, so that in response to head-up tilt the hyperthyroid patients had a more pronounced reduction in systolic BP, and a decrease in diastolic BP compared to an increase in the controls (Fig. 1C and F).

**Figure 2:**
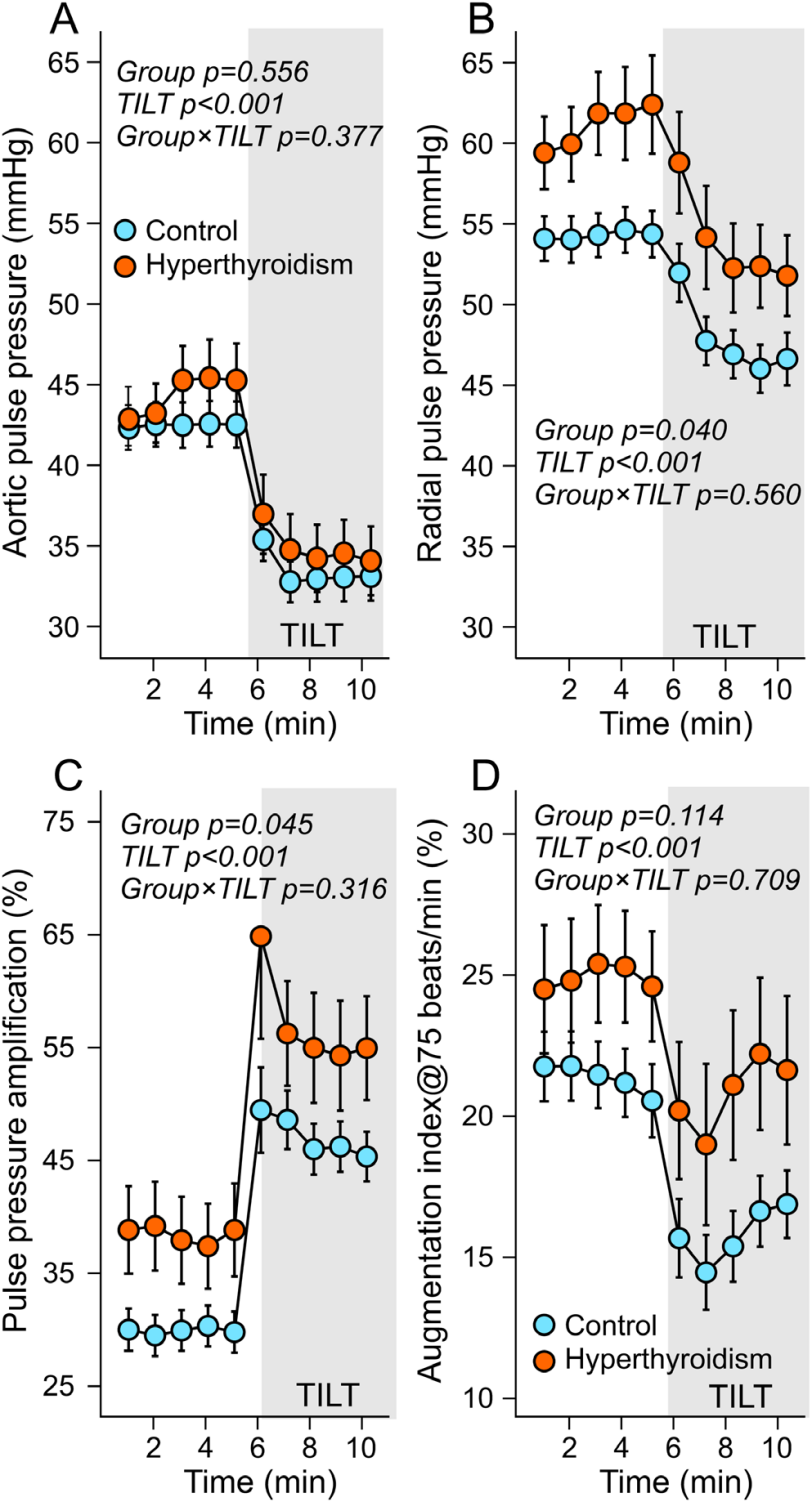
Aortic pulse pressure (A), radial artery pulse pressure (B), pulse pressure amplification (C), and augmentation index adjusted to heart rate 75 beats/min (D) during supine position and passive head-up tilt in 20 patients with hyperthyroidism and in 60 euthyroid controls.

Aortic pulse pressure was similar in both groups (Fig. 2A), but radial artery pulse pressure was increased in hyperthyroidism (Fig. 2B) due to higher pulse pressure amplification (Fig. 2C). The effect of the reflected wave on central pulse pressure, as evaluated using heart rate adjusted AIx (AIx@75), did not significantly differ between the study groups (Fig. 2D). In response to upright posture, pulse pressure and AIx@75 decreased in both groups (Figs. 2A and B).

**Figure 3:**
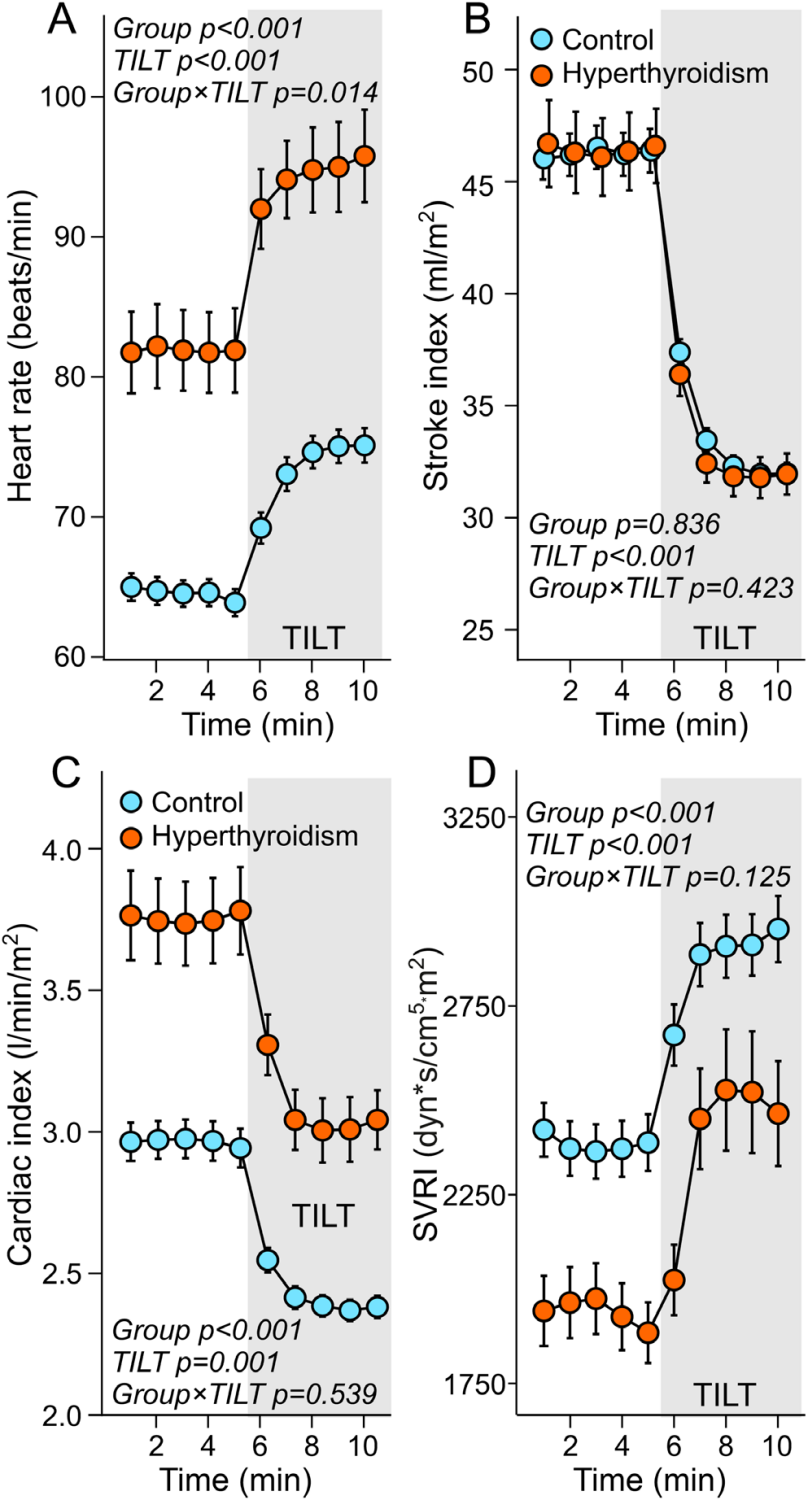
Heart rate (A), stroke index (B), cardiac index (C), and systemic vascular resistance index (SVRI) (D) during supine position and passive head-up tilt in 20 patients with hyperthyroidism and in 60 euthyroid controls.

Whether supine or upright, heart rate was increased in the hyperthyroid patients (Fig. 3A). Heart rate also increased more in response to head-up tilt in the hyperthyroid patients (*P* value 0.014 for posture interaction). Evaluated stroke index was similar in the groups (Fig. 3B), whereas cardiac index was 0.79 (0.16) and 0.71 (0.10) L/min/m^2^ higher in supine position and during head-up tilt, respectively, in the hyperthyroid patients than in the controls (*P* < 0.001, Fig. 3C). SVRI was 425 (112) and 556 (136.0) mm Hg/mL/m^2^ lower in the supine and upright positions, respectively, among hyperthyroid patients than in controls (*P* = 0.002 and <0.001, respectively). Cardiac index and SVRI changed similarly in response to upright posture in both groups (Figs. 3C and D).

**Figure 4:**
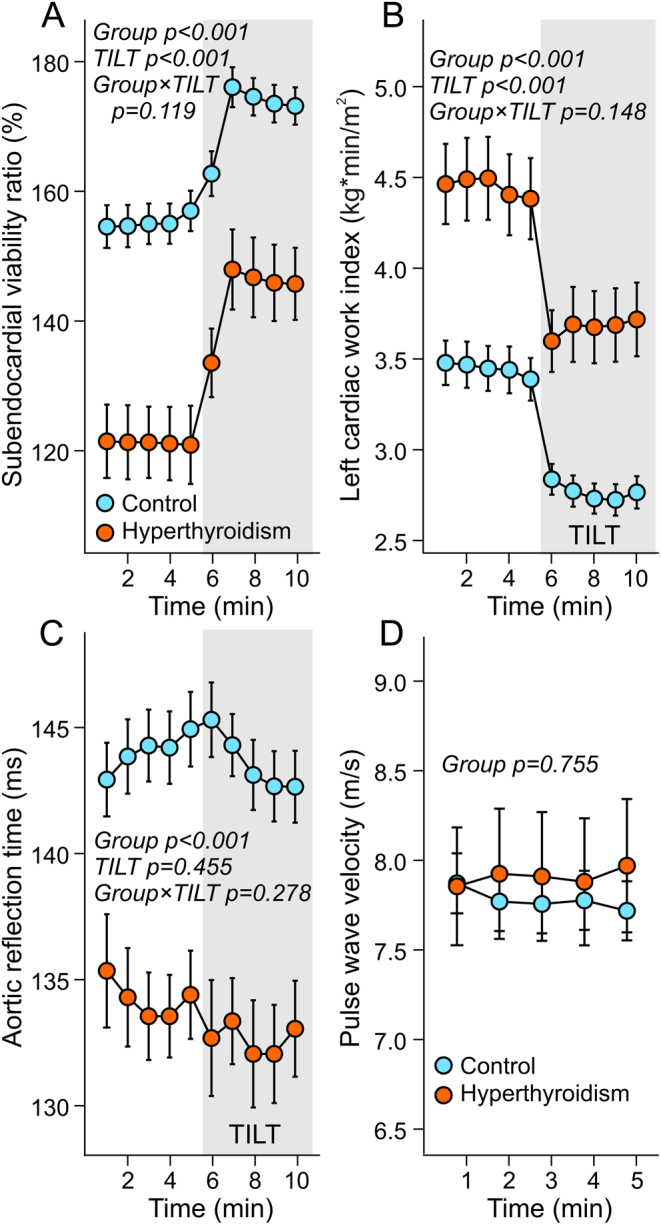
Subendocardial viability (A), left cardiac work index (B), and aortic reflection time (C) during supine position and passive head-up tilt, and supine pulse wave velocity adjusted for mean aortic blood pressure (D) (27) in 20 patients with hyperthyroidism and in 60 euthyroid controls.

The Buckberg index (SEVR) reflecting the balance between time for coronary perfusion and pressure load (28), was reduced by ~19% in hyperthyroidism whether supine or upright (*P* < 0.001) (Fig. 4A). Simultaneously, the work performed by the left ventricle was increased in hyperthyroidism (Fig. 4B). Of note, when the SEVR analyses were adjusted for heart rate, the hyperthyroid and control subjects were no longer significantly different (*P* = 0.131), indicating that the difference in SEVR was due to the higher heart rate in hyperthyroidism. The shortest time for the return of the reflected wave (aortic reflection time) was faster among hyperthyroid patients than in controls, while this variable did not significantly change in response to the head-up tilt (Fig. 4C). Due to a faster left ventricular contraction, the first systolic peak pressure was achieved in 102 (9) ms in the hyperthyroid patients and in 110 (9) ms in the controls (*P* < 0.001). When the aortic reflection time was adjusted for this difference, it was no longer significantly different between the groups (*P* = 0.068). PWV was similar in both study groups (Fig. 4D).

FT4 at diagnosis correlated inversely with aortic reflection time (r_S_ = −0.505, *P* = 0.023), while fT3 during haemodynamic measurements correlated inversely with aortic reflection time (r_S_ = −0.529, *P* = 0.017) and directly with plasma NT-proBNP concentration (r_S_ = 0.566, *P* = 0.012). No other significant correlations between fT3 or fT4 and the haemodynamic variables were detected.

## Discussion

The present study confirms the previous findings of hyperdynamic circulation and reduced systemic vascular resistance in hyperthyroidism. To our knowledge, this study is the first to report altered blood pressure distribution, increased pulse pressure amplification, impaired balance between coronary perfusion time and cardiac pressure load, and elevated left cardiac work in hyperthyroid patients. Furthermore, hyperthyroid patients exhibited more pronounced chronotropy and a greater decrease in peripheral blood pressures in response to head-up tilt compared to euthyroid subjects.

Heart rate and cardiac index were increased in hyperthyroid patients, while stroke index remained unchanged. This indicates that increased heart rate, rather than stroke volume, is a major contributor to the increased cardiac output in hyperthyroid patients. Notably, increased chronotropy is less effective in enhancing cardiac performance than the regulation of preload conditions (6). Our findings contrast with some previous views, as the preload, a major determinant of the stroke volume through the Frank-Starling mechanism, has been suggested to increase in hyperthyroidism due to increased blood volume (1, 6). Increased blood volume would increase venous return to the heart, which, in turn, is a major regulator of the preload (1, 6). This hypothesis stems from a small study conducted by Gibson and Harris in 1939, which demonstrated a modest 5% increase in the blood volume of hyperthyroid patients (29). In contrast, Anthonisen *et al.* reported a slight decrease in total blood volume alongside elevated cardiac output, primarily attributed to an increased heart rate rather than stroke volume, consistent with our findings (30). Some studies have reported unchanged or minute increases in stroke volume and ventricular end-diastolic measures in hyperthyroidism, the findings of which do not support the view of a significant increase in preload in hyperthyroidism (6, 30, 31).

The present finding of increased plasma NT-ProBNP with unchanged NT-ProANP indicates that hyperthyroidism causes cardiac strain without a simultaneous volume overload. ANP is released from secretory granules as a response to cardiomyocyte stretch, and its concentration is greater in the atria than in the ventricles (32). While both atrial and ventricular cardiomyocytes synthesize BNP, it is not stored to the same extent as ANP (32). Under increased pressure, left ventricular BNP synthesis increases, leading to a rise in plasma BNP (33). Altogether, ANP responds more acutely to increases in volume load, whereas higher BNP levels rather reflect sustained cardiac pressure and volume overloads (32). Our results of unaltered stroke index, plasma NT-ProANP, and extracellular water volume indicate that volume overload does not play a major role in the haemodynamics of acute mild-to-moderate hyperthyroidism. In contrast, the 2-fold increase in plasma NT-proBNP in the hyperthyroid patients clearly indicates an increased workload of the heart. Comparable Cornell voltage duration product in the groups may be attributed to the subacute duration of hyperthyroidism.

In the present study, the myocardial perfusion-time to pressure-load relationship assessed by SEVR was decreased simultaneously with an increase in cardiac output and left cardiac work in hyperthyroid patients. SEVR is defined as the diastolic to systolic pressure-time integral ratio, and it estimates the balance between myocardial oxygen supply and demand (34). The decreased SEVR in hyperthyroid patients was attributable to increased heart rate, which can be explained by the shorter diastolic phase of the heart during increased heart rate (13). Haemodynamic changes impairing myocardial perfusion during increased cardiac workload may contribute to the cardiovascular symptoms in hyperthyroidism, such as exercise intolerance, dyspnoea, and chest pain.

The present findings of increased pulse pressure amplification, reduced finger BP, and reduced systemic vascular resistance can be attributed to the vasodilatory effects of thyroid hormones. Previously, thyroid hormones have been reported to activate cell membrane K^+^ channels in smooth muscle and increase nitric oxide production in the endothelium (5, 35, 36). Increased metabolic demand in tissues significantly contributes to the alterations in peripheral haemodynamics and the hyperdynamic circulation in hyperthyroidism (6, 37). Our results showed changes in peripheral haemodynamics in hyperthyroidism. The reduction in finger BPs can be explained by altered thermoregulation, as the hand area is an important heat-exchange organ with arteriovenous anastomoses and dense vascularization (38). Excess heat is radiated through vasodilation when blood flow through the hands is increased via the anastomoses (38). In the fingers, this flow can increase by up to 500% (38, 39).

The present findings of increased pulse pressure amplification, in the absence of significant changes in augmentation index (AIx@75) and pulse wave velocity (PWV), do not support the view of increased arterial stiffness in hyperthyroidism (40). Pulse pressure amplification is the increase in pulse pressure from the aorta to the peripheral arteries (26, 41). This phenomenon is more evident in younger individuals with more elastic central arteries (26, 41). Earlier findings regarding changes in AIx@75 have been inconclusive, reporting both decreased and increased AIx@75 in hyperthyroidism (10, 11, 12, 13). Although AIx@75 is influenced by arterial stiffness, it describes the influences of wave reflection on central pulse pressure (26). In contrast, PWV represents the speed of pulse wave propagation in the vessels, making it a direct measure of arterial stiffness (26). Carotid-femoral (cf-PWV) is considered the gold standard measurement of arterial stiffness, and corresponding to our PWV results, Grove-Laugesen *et al.* did not find changes in cf-PWV during laboratory measurements in hyperthyroidism (10).

The differences between the present study groups in lipid, calcium, and glucose metabolism reflect the known effects of hyperthyroidism (42, 43, 44). The discrepant findings of simultaneous elevations in cystatin C and reductions in creatinine values in hyperthyroidism have been previously documented, but the underlying mechanisms remain obscure (45). Despite elevated erythropoietin, haemoglobin levels are usually normal or even decreased in hyperthyroidism, possibly due to altered iron metabolism, haemolysis and oxidative stress leading to shortened erythrocyte survival (46). The significance of the 2 mmol/L difference in plasma sodium between the present study groups remains unknown, but the values were well within the normal range.

The present study has some limitations. The study had a rather small number of participants, and there is a possibility of a selection bias. Since the DYNAMIC study does not routinely include thyroid hormone measurements, euthyroidism in the control group was assumed based on clinical and haemodynamic evaluation without hormonal determinations. This represents a limitation of the study. Due to the recruitment protocol, the most severe cases of hyperthyroidism were excluded. The measurements were conducted shortly after the initiation of ATD therapy, potentially mitigating the hemodynamic changes. Ethically, delaying the treatment of hyperthyroidism was not feasible. Consequently, the included hyperthyroid patients presented with a mild-to-moderate form of biochemical hyperthyroidism. The results may have been influenced by medications or participants' prevailing medical conditions, although there were no significant differences between the groups. The non-invasive methods utilized mathematical functions to derive the haemodynamic variables. However, the methods have undergone validation against invasive measurements, three-dimensional ultrasound, and tonometric recordings (15, 22, 23, 24, 25). Previously, aortic-to-popliteal PWV determination using whole-body impedance cardiography correlated well with tonometric and Doppler ultrasound measurements of PWV (24, 25). Notably, extracellular water volume evaluation using impedance cardiography successfully detected a 4% increase in patients with primary aldosteronism (47). The strength of this study lies in carefully matching patients and controls by age, sex, BMI, and smoking status, with similar alcohol consumption between the groups.

In conclusion, the hemodynamic profile of hyperthyroidism extends beyond being merely hyperdynamic, including alterations in BP distribution and haemodynamic findings related to vasodilatation. Our results suggest that the hyperthyroid heart undergoes significant stress through various mechanisms, potentially contributing to increased cardiovascular morbidity in individuals with hyperthyroidism.

## Supplementary Materials

Supplementary Table 1. Medications used by 60 controls and 20 hyperthyroid patients, n (%).

## Declaration of interest

The authors declare that there is no conflict of interest that could be perceived as prejudicing the impartiality of the study reported.

## Funding

This work was supported by the Finnish Foundation for Cardiovascular Researchhttp://dx.doi.org/10.13039/100007555 (administrative code T60534), the Sigrid Jusélius Foundation (grant number 230159), the State funding for university-level health research, Tampere University Hospital, Wellbeing services counties of Pirkanmaa and South Ostrobothnia (grant nos 9AB057, 9AC076 and 7310/1051), Finnish Cultural Foundation (grant no. 50161804), the Emil Aaltonen Foundation (grant no. 230195), and the University Consortium of Seinäjoki (UCS2023).

## Author contribution statement

NS, SM, PJ, and IP designed the study. JM, EM, and IP (lead) developed methodology. NS, SM, and IP performed investigations. NS and IP performed the statistical analyses and wrote the first draft of the manuscript. All authors reviewed and edited the manuscript and accepted the final version to be submitted.
