# Supplementary material for: Haemodynamics of hyperthyroidism: increased cardiac work and findings related to vasodilatation
Source: Eur Thyroid J. 2024 Oct 24;13(5):e240090. doi: 10.1530/ETJ-24-0090 (PMC11558968; doi:10.1530/ETJ-24-0090)
Supplement: Supplementary Table 1. Medications used by 60 controls and 20 hyperthyroid patients, n (%). [file supplementary_table_1.pdf]

Supplementary Table 1. Medications used by 60 controls and 20 hyperthyroid patients, n (%).

Medication	Control	Hyperthyroidism	p-value
Female hormones (contraception or replacement)	16 (27)	6 (30)	0.772
Calcium channel blocker	6 (10)	0 (0)	0.328
Antihistamine	5 (8)	0 (0)	0.324
Statin	5 (8)	0 (0)	0.324
Thiazide diuretic	4 (7)	1 (5)	1.000
Angiotensin-converting enzyme inhibitor	3 (5)	1 (5)	1.000
Angiotensin receptor blocker	2 (3)	1 (5)	1.000
Beta blocker	2 (3)	1 (5)	1.000
Levothyroxine	3 (5)	0 (0)	0.569
Acetylsalisylic acid	2 (3)	0 (0)	1.000
Inhaled glucocorticoid	2 (3)	0 (0)	1.000
Metformin	0 (0)	2 (10)	0.060
Sedative	2 (3)	0 (0)	1.000
Proton pump inhibitor	0 (0)	2 (10)	0.060
NSAID or paracetamol	0 (0)	1 (5)	0.250
Alpha + beta blocker	1 (2)	0 (0)	1.000
Potassium sparing diuretic	1 (2)	0 (0)	1.000
Ezetimibe	1 (2)	0 (0)	1.000
Dipeptidyl peptidase-4 inhibitor	1 (2)	0 (0)	1.000
Alfuzosin	1 (2)	0 (0)	1.000
Amitriptyline	1 (2)	0 (0)	1.000
Cyclosporine	0 (0)	1 (5)	0.250
Escitalopram	1 (2)	0 (0)	1.000
Finasteride	1 (2)	0 (0)	1.000
Glucosamine	1 (2)	0 (0)	1.000
Mefloquine	1 (2)	0 (0)	1.000
Moxonidine	1 (2)	0 (0)	1.000
Prednisolone	0 (0)	1 (5)	0.250
Tamoxifen	1 (2)	0 (0)	1.000
Varenicline	1 (2)	0 (0)	1.000

Abbreviations: NSAID, non-steroidal anti-inflammatory drug.
